# Mindfulness-Based Cognitive Therapy for Unmedicated Obsessive-Compulsive Disorder: A Randomized Controlled Trial With 6-Month Follow-Up

**DOI:** 10.3389/fpsyt.2021.661807

**Published:** 2021-08-03

**Authors:** Tianran Zhang, Lu Lu, Fabrizio Didonna, Zhen Wang, Haiyin Zhang, Qing Fan

**Affiliations:** ^1^Shanghai Mental Health Center, Shanghai Jiao Tong University School of Medicine, Shanghai, China; ^2^Department of Medical Psychology, Xinhua Hospital, Affiliated to Shanghai Jiao Tong University School of Medicine, Shanghai, China; ^3^Casa di Cura Villa Margherita, Vicenza, Italy; ^4^Shanghai Key Laboratory of Psychotic Disorders, Shanghai, China

**Keywords:** MBCT, mindfulness, obsessive-compulsive disorder, randomized controlled trial, psychotherapy

## Abstract

**Background:** This was the first randomized controlled trial (RCT) designed to compare the efficacy of mindfulness-based cognitive therapy (MBCT) on unmedicated obsessive-compulsive disorder with that of the first-line treatment for OCD (SSRIs) or a placebo, as well as to analyze the treatment acceptability and safety of MBCT.

**Methods:** A total of 123 unmedicated OCD patients with mild to moderate symptoms were randomly assigned into selective serotonin reuptake inhibitors group (SSRIs group), MBCT group or psycho-education group (PE group), respectively. They were intervened for 10 weeks. The Yale–Brown Obsessive-Compulsive Scale (Y-BOCS) grade was the primary outcome, and Hamilton Depression Scale-24 (HAMD-24) and Hamilton Anxiety Scale (HAMA) grades were secondary outcomes to be measured at baseline, mid-intervention, post-intervention and 14, 22, and 34 weeks of follow-up. The Five Facet Mindfulness Questionnaire (FFMQ) and Sheehan Disability Scale (SDS) were used to assess mindfulness and social functions, respectively. In addition, treatment acceptability (dropout rate and frequency of occurrence) and safety [adverse event (AE)] of MBCT were investigated.

**Results:** Significant differences were detected in the treatment responses among SSRIs group, MBCT group and PE group. Notably, treatment responses were significantly better in the former two groups than that of PE group (χ^2^ = 6.448, *p* = 0.04), although we did not identify significant differences between SSRIs group and MBCT group (χ^2^ = 1.220, *p* = 0.543). Observed until 6 months of follow-up, there were no significant differences in treatment response among three groups. No AE was recorded in MBCT group.

**Conclusion:** MBCT is effective in the treatment of unmedicated OCD with mild to moderate symptoms comparable to that of SSRIs, which contributes to maintain the treatment outcomes at follow-up. Besides, MBCT is safe with a good clinical compliance.

## Introduction

Obsessive-compulsive disorder (OCD) is a psychiatric condition characterized by repeated obsessions and compulsions (1), which causes chronic damage to cognitions, social functions and quality of life (2). In mainland China, the current and lifetime prevalence of OCD from 2013 to 2015 were 1.60 and 2.40%, respectively (3).

Selective serotonin reuptake inhibitors (SSRIs) and cognitive-behavioral therapy (CBT) with exposure and response/ritual prevention (ERP) are now the first-line treatments for OCD, which have been developed for a long time. However, a part of OCD patients develop resistance to them, and their limitations also trigger us to develop more effective treatments of OCD. It is reported that 25% of OCD patients refuse to use CBT-ERP; 20% drop out the treatment; and 15–40% are poorly responded (4–6). In another clinical trial, 30–40% of OCD patients treated with CBT, SSRIs or a combination develop residual symptoms (7).

Mindfulness treatment has emerged as a novel and promising approach for the treatment of OCD. Mindfulness is a mental state and process leading to a non-judgmental awareness of present moment experience (8), and a severe deficiency of mindfulness is considered to be a feature of OCD (e.g., attentional bias, rumination, thought–action fusion, inflated responsibility, self-invalidation of sensory experience, self-distrust) (9, 10). It is believed that mindfulness-based treatment is beneficial to the following aspects: (1) To integrate a less frightening ERP intervention with an anti-ruminative and anti-avoidant attitude (mindful exposure) that reduces the risk of dropout; (2) To improve insight, reality testing and acceptance attitude to experience the impermanence of thoughts, emotions and sensations; (3) To develop metacognitive and defusion, decentering and disidentification processes; (4) To strengthen the sense of responsibility, self-trust, self-compassion and self-forgiveness; (5) To weaken the obsessive cognitive biases, dysfunctional beliefs and compulsive behavior of OCD. So the MBCT for OCD program is trying to help patients to develop a better relationship with their obsession and find a new response to the harmless context instead of the compulsion.

The standard CBT is aimed to change the cognitive content, but the aim of mindfulness-based intervention is to change the approach of relating to the cognitive content. Similar to conventional interventions, mindfulness-based cognitive therapy (MBCT) improves specific symptoms or intrusive thoughts in OCD patients. Notably, MBCT highlights to develop a different mental style in OCD patients and changes the relationship between OCD patients and their symptoms. Thus, through neutralizing cognitive biases and mechanisms, altering affect regulation systems and dysfunctional responses, MBCT contributes to change a series of symptoms stimulated by harmless triggering stimuli in OCD patients.

The manualized treatment program used in this study (11) basically satisfied to the structure of the original MBCT model generated by Segal et al. (12), but it has been modified to a 10-week treatment to address the unique clinical features of OCD. Key features of this program includes normalizing obsessive experience, developing trust and self-validation, cognitive intervention and intensive training in mindfulness and self-compassion. In a pilot study, Didonna et al. (10) showed a significant improvement in Yale–Brown Obsessive-Compulsive Scale (Y-BOCS) scores and other clinical measures, and a correlated improvement in mindfulness skills in OCD patients after MBCT intervention. As a well-recognized treatment, MBCT is proved to be helpful in many mental disorders, such as major depression disorder, panic disorder, social phobia, etc., and there have been many meta-analysis studies reported the effectiveness of MBCT (13–18). Likewise, the rationale and utility of MBCT in the treatment of OCD has been demonstrated.

Most of the existing research about MBCT intervention in OCD patients is preliminary, with a small sample size (19, 20) and qualitative data (21, 22), which suggested that MBCT can potentially have a positive therapeutic effect on predisposing, activating and maintaining factors of OCD. And the only one randomized controlled trial (RCT) with a large sample size (*n* = 125) investigated the effectiveness of MBCT as a complementary treatment option for OCD patients who are poorly responded to CBT (23). In this research, participants were randomized to either an MBCT group or to a PE group for eight 2-h sessions interventions. The finding suggested that, compared to a psychoeducational program, MBCT leads to accelerated improvement of self-reported OC symptoms and secondary outcomes, but not of clinician-rated OC symptoms. In fact, in addition to being a synergistic intervention for patients with residual symptoms, mindfulness-based treatments like MBCT has been proven to effectively alleviate acute anxiety and depression (24). Leeuwerik et al. (25) investigated two qualitative thematic analysis of interview data obtained from participants in a mindfulness-based ERP course and a MBCT course adapted for OCD without ERP tasks. Three common main themes emerge in both MB-ERP and MBCT for OCD and some different benefits are obtained from these two mindfulness-based interventions (MBIs). So, we adapted most of the valid elements into the MBCT, including the mindful exposure in the present study, which is different from previous programs.

Hence, unlike existing preliminary researches in which MBCT is applied as a anti-depression program or a synergistic intervention, this study was aimed to be the first equivalent clinical trial designed to test the efficacy, treatment acceptability and safety of MBCT on OCD patients, and compared with those of the first-line treatments SSRIs or a placebo for unmedicated OCD with mild to moderate symptoms. We hypothesized that the efficacy of MBCT on alleviating obsession, compulsion, anxiety and depression symptoms of OCD patients is equal to SSRIs and better than PE, and the MBCT for OCD program can improve mindfulness level and adherence to treatment of OCD patients.

## Methods

### Design

**Figure 1:**
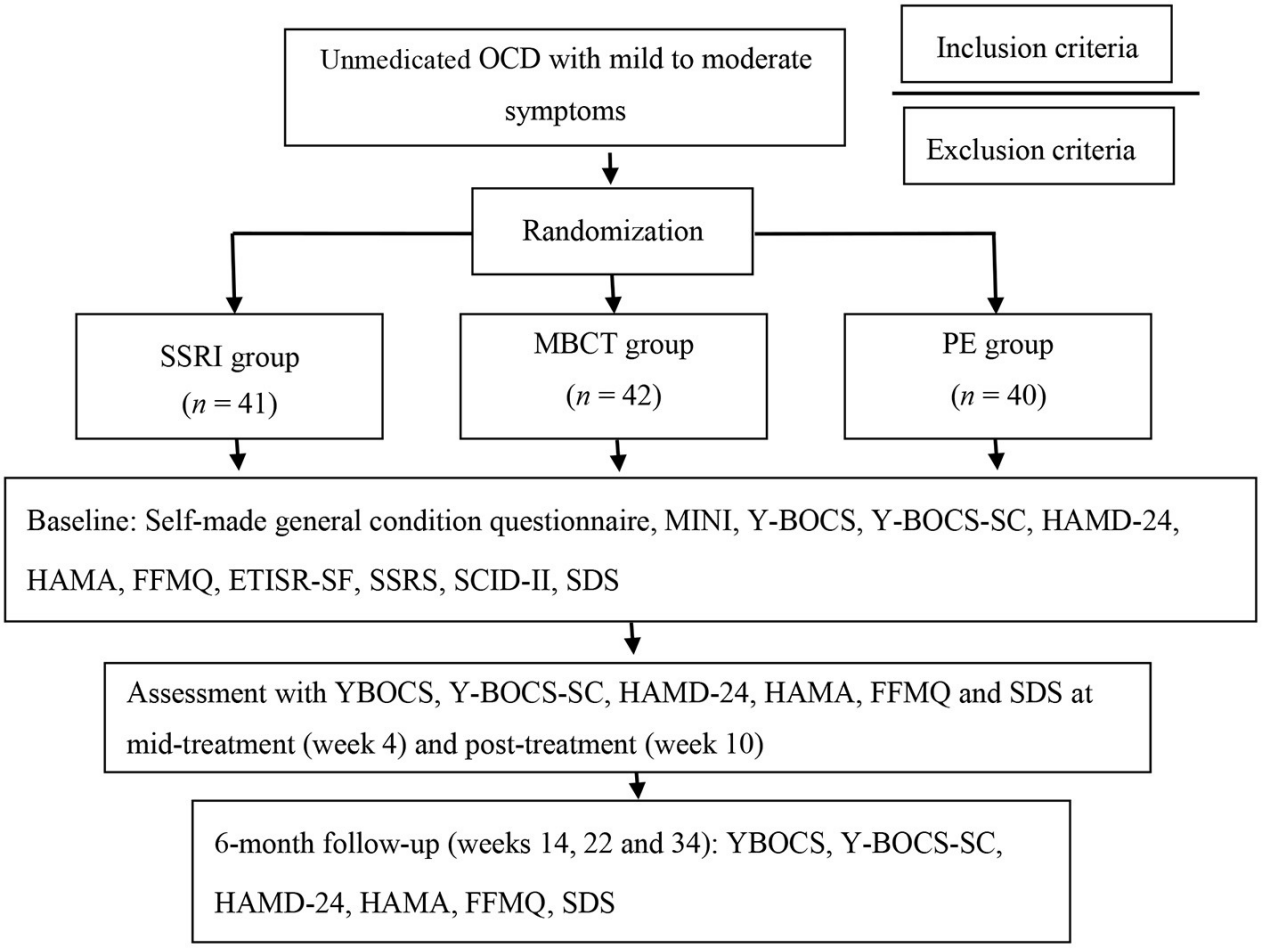
Flow chart. MINI, Mini-International Neuropsychiatric Interview; Y-BOCS, Yale–Brown Obsessive Compulsive Scale; Y-BOCS-SC, Yale–Brown Obsessive-Compulsive Scale Symptom Checklist; HAMD-24, Hamilton Depression Scale-24; HAMA, Hamilton Anxiety Scale; FFMQ, Five Facets of Mindfulness Questionnaire; ETISR-SF, Early Trauma Inventory Self Report, Short Form; SSRS, Social Support Rating Scale; SCID-II, Structured Clinical Interview for DSM-IV Axis II disorders; SDS, Sheehan Disability Scale.

As shown in Figure 1, this was an single-blind, randomized, actively controlled clinical trial with three study arms: SSRI group; MBCT group; and PE group (26). Eligible unmedicated OCD participants, including those without a medication history of psychiatric drugs, or have been discontinued for more than 8 weeks were recruited from 7th June, 2017 to 30th July, 2018. They were assigned to three groups using a pre-determined random table generated by Microsoft Excel 2010. Only the case manager had the information of the randomization. The numbers of the participants were not decoded until the intervention group was assigned. OCD patients were intervened for 10 weeks and followed up. The first participant ended the 6-month follow-up on 10th March, 2018, and the final follow-up was on 8th May, 2019. Primary and secondary outcomes were assessed at the following time points: Week 0 (baseline), week 4 (mid-treatment), week 10 (post-treatment) and week 14, 22, and 34 (the follow-up phase). The study was approved by the Ethics Committee of the Shanghai Mental Health Center (SMHC), and registered on ClinicalTrials.gov by the SMHC on 14th May 2017 (NCT03179839).

### Participants

Participants were recruited from the outpatient department of the SMHC. Informed consent was obtained prior to the clinical trial, and participants of 3 groups were blind of the group, but clear about the possible side effect from psychotherapy and medication. All participants were free to withdraw consent and leave the trial at any time. Inclusion criteria: (1) Male or female OCD patients aged 18–54 years who were diagnosed by a Chinese version of the Mini-International Neuropsychiatric Interview [MINI] for DSM-5 with mild and moderate level of OCD symptoms, which means Y-BOCS score ranging from 12 to 25 grades (27); (2) Junior/middle school education or above; (3) Lack of a history of any psychiatric medication or, discontinued for 8 weeks before the requirement; (4) Sufficient visual and acoustic ability to complete the examination and questionnaires for the study; (5) Informed consent was obtained from both patients and their guardians. Exclusion criteria: (1) Other psychiatric disorders diagnosed by DSM-5 Axis I diagnostic criteria; (2) Severe physical or central nervous system disease; (3) High negative self-concept or high risk of suicide; (4) Substance abuse problems, pregnant women or plan to be, and lactating women; (5) Severe obsessive and compulsive symptoms; (6) Involved in other concurrent psychological therapy; (7) History of mindfulness-based interventions without significant effects.

**Figure 2:**
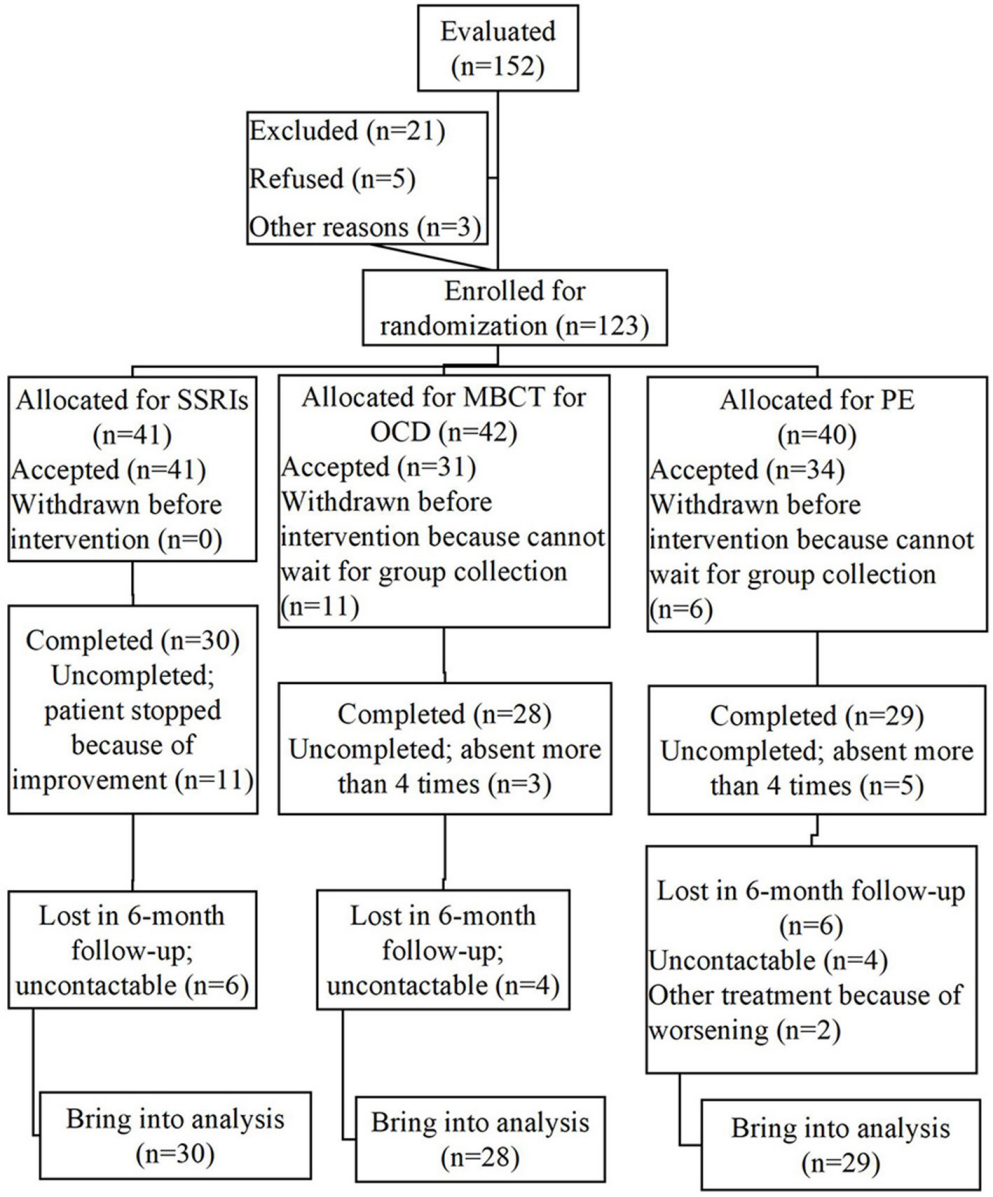
CONSORT flowchart.

The program “G^*^Power” conducts a power analysis (28). According to the theoretical considerations, results of comparable studies (29) and pilot data, it is assumed that the priori test power 1 − β = 0.8, while the effect size of *d* is 0.8. A minimum sample size of *n*1 = *n*2 = *n*3 = 26 is sufficient to detect this effect through a *t*-test at the significance level of *p* < 0.05 for a group × time interaction. Considering the dropout rate, a total of 123 eligible OCD patients were finally recruited from June 2017 to August 2018 in the outpatient department of SMHC. Before interventions, 11 participants in MBCT group and six in PE group were dropped out because of the waiting period for collection and involvement in other treatments for security guarantee. Hence, there were 41, 31, and 34 participants in SSRI group, MBCT group and PE group, respectively. At the post-treatment assessment, 19/106 (17.92%) cases dropped out, involving 11, 3, and 5 in SSRI, MBCT and PE groups, respectively (percentage of attendance < 0.6 was defined as dropout). The follow-up rate for 1, 3, and 6 months were 74.53, 67.92, and 66.98%, respectively. Inclusion and exclusion details were shown in Figure 2. There were no significant differences in demographic characteristics and baseline data between OCD patients who completed the treatment and dropout cases.

### Interventions

Interventions in the three groups were conducted in the outpatient department of SMHC. Both MBCT and PE program consist of 11 sessions with 150 min each for 10 weeks, and each group included 6–8 OCD patients with two therapists.

MBCT intervention in OCD patients was conducted according to the manual (10) drawn from the MBCT for depression program (12), but adapted to OCD with different rationale and several unique practices specifically tailored to OCD features (10, 30). Briefly, MBCT for OCD includes a total of 11 sessions: Session 1 is aimed at describing the concept of mindfulness and recognizing the tendency of our minds to wander. Session 2 is focused on understanding the relationship between OCD and mindfulness. Session 3 is aimed at teaching family members how to help and support patients effectively. Session 4 is about understanding mistrust and its role in OCD so that patients can develop self-trust. In Session 5, patients are trained in validating and using their senses to prevent the distorted cognitive mechanisms that lead to obsessive mechanisms. Session 6 is aimed at helping patients understand their relationship with thoughts and develop the ability of decentering and disidentification. The focus of Session 7 is on developing acceptance toward internal experience without judgement, interpretations, and reactions. The theme of Session 8 is mindful exposure, and Session 9 is about self-compassion and self-forgiveness. The focus of Session 10 is learning to take constructive risks in a mindful way. Session 11 is a 1-day mindfulness retreat, aimed at reviewing previous practices and enhancing motivation to continuing the practices (26). During each session, participants received handouts to facilitate comprehension. Participants were encouraged to practice mindfulness exercises for at least 1 h a day at home after each session with audio tracks.

The PE program, as an active placebo control condition, consisted of propagating information about OCD, including epidemiology, biological and psychosocial causes, clinical symptoms, family burden of OCD, and supportive sharing and discussion (29, 31). The theme of Session 1 is the formation of the group, including establishing rules and familiarizing the members with each other. The theme of Sessions 2 and 3 is the introduction to pathogenesis and influencing factors of OCD. Session 4 is aimed to educate the family members of OCD patients on how to help their families and themselves. Sessions 5 and 6 is about to provide information on the treatment of OCD. The goals of Sessions 7 and 8 are for patients to share their own experiences with OCD and to discuss their experience with treatment. The theme of Sessions 9 and 10 is the prevention and relapse of OCD. And Session 11 is to discuss plans for the future and the support of each other (26).

This control group can choose to use SSRI drugs approved by the State Food and Drug Administration (SFDA) for the treatment of OCD (Sertraline, Fluvoxamine, initial dose of 50 mg). Participants could adjust the dosage of SSRIs once a week at most within the limit of the maximum dose in the instructions of psychiatrists from this study. Benzodiazepine drugs could also be medicated in OCD patients with sleep disorders, but may not be continuously used for over 2 weeks. Other psychotropic drugs were prohibited. The drugs in this study are commonly used drugs with good safety profiles and common adverse reactions including dry mouth, constipation, nausea, indigestion, dizziness, fatigue, and sweating (32).

### Treatment Fidelity

The instructors for MBCT were the same to those for PE: Psychotherapists or psychiatrists specialized to OCD treatment; Trained by the founder of MBCT for OCD (Fabrizio Didonna) and one of the founders of MBCT (Mark Williams) to ensure treatment fidelity. Supervisions once a month during the study were provided by Dr. Didonna to ensure the treatment fidelity as well. Psychiatrists who prescribed SSRIs attended regular meetings to make sure that medication use consistently fitted the research requirements. We also have the quality control supervisors to check the consistency and validity of the research regularly.

### Measurements

All assessments were carried out by qualified psychological physicians who were trained before the program and had evaluator consistency meetings once a month for quality control. All of them were blind for the intervention group of participants from the beginning to the last assessments.

Y-BOCS scores (33) were the primary outcome for evaluating the effectiveness. According to the international consensus criterion (34), improvement of the Y-BOCS scores ≥ 35% (reduction rate ≥ 35%) is defined as a treatment response. Partial response and non-response are defined as a 25–35% reduction rate and <25% reduction, respectively.

Hamilton Depression Scale-24 [HAMD-24: (35)] and the Hamilton Anxiety Scale scores [HAMA: (36)] were the secondary outcomes, which reflected depression and anxiety. Also, the Five Facet Mindfulness Questionnaire [FFMQ: (37, 38)] and the Sheehan Disability Scale [SDS: (39)] were used to assess mindfulness level and social functions of OCD patients, respectively.

Dropout rate and frequency of occurrence were recorded to evaluate treatment acceptability, whereas adverse events (AEs) were the treatment safety indicators.

### Statistical Analyses

An overall analysis in all participants, involving those dropped out in SSRIs group because of improved symptoms and those absent in several sessions of MBCT and PE, may lead to an underestimated efficacy of SSRIs. Furthermore, we only performed a per-protocol analysis in participants who completed all sessions of interventions for assessing the efficacy due to the equivalence of SSRIs and MBCT in the treatment of OCD.

An analysis of variance (ANOVA) test and Chi-square test were performed to compare continuous variables, and categorical variables, respectively. Multiple imputations were conducted to estimate the scores for non-follow-up participants after post-treatment. The response to the Y-BOCS was analyzed by the rank sum test, whereas the comparisons at different time points were conducted by ANOVA for repeated measurements of baseline HAMA and HAMD scores as covariates. A two-sided *p* value < 0.05 was considered statistically significant, which was also available for the secondary analysis. Corresponding 95% confidence intervals (95% CI) were calculated whenever possible.

## Results

### Baseline Characteristics

There was a significant difference in gender of the total 87 participants, which including 60 male and 27 female (χ^2^ = 12.517, *p* < 0.001), and no difference in age (AVG = 28, *t* = 0.550, *p* = 0.584). As shown in Table 1, no significant differences in demographic characteristics or Y-BOCS scores were found among three groups. However, baseline HAMD and HAMA scores differed among three groups even after randomization. Bonferroni correction data showed that HAMA scores (*p* = 0.001) and HAMD scores (*p* = 0.004) were significant lower in PE group than those of SSRIs group. No significant differences were found in the other psychometric variables, including FFMQ and SDS.

**Table 1:** Baseline characteristics of the participants.

**Variable**	**Mean** **±** **SD or Rate% (** ***n*** **)**				
	**SSRIs (*N* = 30)**	**MBCT (*N* = 28)**	**PE (*N* = 29)**	***F*/***χ**^2^***	***p***
Gender				1.136	0.567
Male	70% (21)	75% (21)	62.1% (18)		
Female	30% (9)	25% (7)	37.9% (11)		
Age at enrollment	27.43 ± 6.87	29.39 ± 7.37	28.45 ± 6.32	0.592	0.555
Education (total years)	14.83 ± 2.39	15.68 ± 1.81	15.59 ± 2.35	1.298	0.279
Mental disorder history				4.156	0.385
No	50% (15)	39.3% (11)	31.0% (9)		
OCD	50% (15)	53.6% (15)	65.5% (19)		
Other	0.0% (0)	7.1% (2)	3.4% (1)		
Treatment history				8.173	0.226
No	72.4% (21)	46.4% (13)	69.0% (20)		
Western medicine	20.7% (6)	35.7% (10)	27.6% (8)		
Chinese medicine	0.0% (0)	7.1% (2)	0.0% (0)		
Psychotherapy	6.9% (2)	10.7% (3)	3.4% (1)		
Current state				1.246	0.870
Acute	16.7% (5)	7.1% (2)	13.8% (4)		
Subacute	13.3% (4)	14.3% (4)	13.8% (4)		
Chronic	70.0% (21)	78.6% (22)	72.4% (21)		
First onset age	19.21 ± 6.67	20.89 ± 8.42	21.72 ± 5.88	0.958	0.388
Total disease course	8.48 ± 6.88	8.55 ± 6.13	6.99 ± 5.59	0.568	0.569
Family history				2.145	0.342
Negative	75.9% (22)	85.7% (24)	89.7% (26)		
Positive	24.1% (7)	14.3% (4)	10.3% (3)		
Y-BOCS	21.03 ± 3.53	21.18 ± 3.31	19.79 ± 3.70	1.353	0.264
HAMD	16.67 ± 8.21	12.39 ± 5.91	10.03 ± 5.57	7.451	0.001
HAMA	11.63 ± 6.64	9.29 ± 5.78	7.14 ± 4.80	4.437	0.015
FFMQ	109.60 ± 8.48	110.07 ± 11.58	111.86 ± 11.10	0.382	0.684
SDS	15.60 ± 6.51	15.46 ± 5.97	12.90 ± 6.88	1.610	0.206

### Treatment Response

As assessed for primary outcomes at post-treatment, treatment response was significantly different in the three groups compared with baseline levels (Table 2). Notably, treatment responses were significantly better in the former two groups than that of PE group (χ^2^ = 6.448, *p* = 0.04), although we did not identify significant differences between SSRIs group and MBCT group (χ^2^ = 1.220, *p* = 0.543). After the 6 months of follow-up, there were no significant differences in treatment responses among three groups.

**Table 2:** Non-parametric test results of treatment response.

		**Percentage (** ***N*** **)**			**Std**.	***p***
**Time**	**Group**	**Response**	**Partial response**	**Non-response**		
Pre to post	SSRIs	43.3% (13)	26.7% (8)	30.0% (9)	−2.059	0.040
	MBCT	39.3% (11)	17.9% (5)	42.9% (12)		
	PE	27.6% (8)	10.3% (3)	62.1% (18)		
Pre to 6-month	SSRIs	47.8% (18)	10.0% (3)	30.0% (9)	−0.099	0.921
follow-up	MBCT	52.4% (18)	14.3% (4)	21.4% (6)		
	PE	58.6% (17)	10.3% (3)	31.0% (9)		

### Efficiency of Treatment

Using repeated measures ANOVA with the HAMA and HAMD scores at baseline as covariates, Y-BOCS, Y-BOCS-O, Y-BOCS-C, HAMD, HAMA, FFMQ, and SDS scores were found significantly elevated in all three groups (Table 3). In addition, there were no significant differences in these scores at post-treatment and 6 months of follow-up. A significant result in HAMD scores was yielded from the interaction effect of time point and intervention.

**Table 3:** Results of descriptive statistics by condition (mean ± SD).

	**Group**	***N***	**Pre**	**4 weeks**	**Post (10 weeks)**	**1-month follow-up**	**3-month follow-up**	**6-month follow-up**
Y-BOCS	SSRIs	30	21.03 ± 3.5	15.33 ± 5.5	12.52 ± 6.9	12.32 ± 6.9	12.42 ± 6.8	13.35 ± 5.8
	MBCT	28	21.18 ± 3.3	15.61 ± 4.8	13.54 ± 6.1	13.36 ± 4.7	14.36 ± 4.2	13.66 ± 4.5
	PE	29	19.79 ± 3.7	16.76 ± 4.7	14.97 ± 4.0	12.79 ± 4.8	13.57 ± 4.7	11.74 ± 6.0
-O	SSRIs	30	11.07 ± 1.9	7.83 ± 2.7	6.81 ± 3.6	6.59 ± 3.8	6.52 ± 3.5	6.72 ± 2.8
	MBCT	28	11.00 ± 1.5	7.71 ± 2.4	7.07 ± 3.3	6.97 ± 2.7	7.48 ± 2.7	6.98 ± 2.6
	PE	29	10.38 ± 2.6	8.66 ± 2.5	7.76 ± 2.1	6.89 ± 2.3	6.77 ± 2.4	5.83 ± 3.0
-C	SSRIs	30	9.97 ± 2.7	7.50 ± 3.1	5.72 ± 3.6	5.73 ± 3.5	5.90 ± 3.6	6.63 ± 3.1
	MBCT	28	10.21 ± 2.3	7.89 ± 2.8	6.46 ± 3.1	6.39 ± 2.4	6.88 ± 1.8	6.6 ± 2.2
	PE	29	9.41 ± 2.1	8.10 ± 2.6	7.21 ± 2.8	5.90 ± 3.6	6.80 ± 2.7	5.91 ± 3.3
HAMD	SSRIs	30	16.67 ± 8.2	9.20 ± 6.8	7.62 ± 5.8	6.70 ± 4.6	7.18 ± 4.4	7.15 ± 4.1
	MBCT	28	12.39 ± 5.9	10.43 ± 6.1	9.18 ± 6.7	6.92 ± 3.9	9.50 ± 6.3	6.09 ± 4.1
	PE	29	10.03 ± 5.6	7.52 ± 5.0	9.38 ± 6.1	6.01 ± 5.4	7.00 ± 5.0	5.37 ± 3.8
HAMA	SSRIs	30	11.63 ± 6.6	5.87 ± 5.7	5.37 ± 5.0	4.73 ± 3.8	4.75 ± 3.8	4.79 ± 3.5
	MBCT	28	9.29 ± 5.8	8.00 ± 4.9	6.18 ± 4.6	4.94 ± 2.5	5.95 ± 3.6	4.06 ± 2.3
	PE	29	7.14 ± 4.8	5.28 ± 3.8	6.48 ± 4.5	3.72 ± 3.3	4.65 ± 3.4	3.41 ± 3.0
FFMQ	SSRIs	30	109.60 ± 8.5	113.10 ± 130.5	113.80 ± 10.6	115.20 ± 10.9	116.87 ± 9.8	118.08 ± 10.0
	MBCT	28	110.07 ± 11.6	116.07 ± 13.5	119.40 ± 14.7	119.75 ± 11.3	119.80 ± 10.7	121.69 ± 12.1
	PE	29	111.86 ± 11.1	114.21 ± 14.4	112.14 ± 21.4	119.17 ± 13.1	116.86 ± 11.5	120.43 ± 8.9
SDS	SSRIs	30	15.60 ± 6.5	11.07 ± 5.8	9.26 ± 6.5	9.69 ± 6.6	10.39 ± 4.6	9.31 ± 5.6
	MBCT	28	15.46 ± 6.0	11.50 ± 4.8	9.39 ± 5.6	10.00 ± 3.3	9.86 ± 3.0	9.13 ± 3.8
	PE	29	12.90 ± 6.9	12.03 ± 5.7	12.48 ± 14.9	9.66 ± 3.7	10.79 ± 3.6	9.45 ± 4.0

*-O, obsession subscale of the Y-BOCS; -C, compulsive subscale of the Y-BOCS*.

### Treatment Acceptability

During the group treatment, there was a significant difference in the frequency of occurrence between MBCT (0.87 ± 0.218) and PE group (0.74 ± 0.217) (*F* = 6.620, *p* = 0.012), indicating a better compliance to MBCT than that of PE (Table 4).

**Table 4:** Results of ANOVA of repeated measures between conditions.

	**Pre to post**						**Pre to 6-month follow-up**					
**Outcome measure**	**Time effect**		**Condition effect**		**Interaction effect**		**Time effect**		**Condition effect**		**Interaction effect**	
	***F***	***p***	***F***	***p***	***F***	***p***	***F***	***p***	***F***	***p***	***F***	***P***
Y-BOCS	14.947	<0.001	0.802	0.452	1.898	0.113	9.265	<0.001	0.478	0.623	1.327	0.216
-O	16.391	<0.001	0.605	0.549	1.834	0.125	11.184	<0.001	0.800	0.455	1.909	0.044
-C	8.162	<0.001	0.851	0.431	1.357	0.251	5.113	<0.001	0.199	0.820	0.652	0.768
HAMD	23.140	<0.001	1.505	0.228	7.110	<0.001	36.662	<0.001	1.310	0.275	4.600	<0.001
HAMA	19.765	<0.001	1.194	0.308	5.352	0.001	32.082	<0.001	1.327	0.271	3.580	0.002
FFMQ	7.282	0.001	0.567	0.569	2.010	0.102	16.646	<0.001	0.827	0.441	1.281	0.257
SDS	13.621	<0.001	0.053	0.948	2.646	0.052	14.634	<0.001	0.055	0.947	1.542	0.175

In the 10-week treatment, 11/41 (26.83%) cases in SSRIs group dropped out due to drug withdrawal, 3/31 (9.68%) in MBCT group dropped out due to absent for more than four times (one was too busy to attend, one left the city, and one for personal reasons); and 5/34 (14.71%) in PE group dropped out due to absent for more than four times (all for personal reasons). There were 16 participants who did not complete the 6-month follow-up, involving 6, 4 and 6 cases in SSRIs, MBCT PE group, respectively. The majority was loss of follow-up, and only two cases in PE reported AEs. The dropout rate was comparable at post-treatment or follow-up without a significant difference.

### Treatment Safety

There were two PE participants who were modified to other treatments, involving one received SSRIs and the other received psychotherapy in the follow-up, because of their subjective will for worsening symptoms and requested for another treatment. Also, the assessment result of increased Y-BOCS scores was managed as an AE. There was no AE reported in MBCT group. No adverse reactions of SSRIs were recorded as well.

### Investigations

FFMQ score of participants in SSRI and PE groups also increased. We found a significant correlation between the Y-BOCS scores and FFMQ scores at both post-treatment and 6 months of follow-up (Table 5).

**Table 5:** Correlations between response results and other variables.

	**Variables**	**Coefficient**	***p***
Pre to post	FFMQ increasing score	*N* = 87 *r* = 0.215	0.045
	Groups of MBCT for OCD	*N* = 28 *X^2^* = 9.968	0.126
Pre to follow-up	FFMQ increasing score	*N* = 87 *r* = 0.235	0.029
	Groups of MBCT for OCD	*N* = 28 *X^2^* = 7.606	0.268

Furthermore, OCD patients in MBCT group were subclassified to four groups according to different group members and therapists for subgroup analysis. Y-BOCS response did not significantly correlated to the four subgroups, showing that different group members and therapists did not affect the validity of the MBCT in the treatment of OCD.

## Discussion

This was a prospective RCT involving three arms. Unmedicated OCD patients with mild to moderate symptoms presented a better response to Y-BOCS in MBCT and SSRIs groups than those in PE group, although the significant difference disappeared at the 6-month follow-up. Furthermore, MBCT presented a good treatment compliance in OCD patients over the other treatments.

In the present study, the short-term effectiveness of MBCT on OCD was consistent with most previous findings (10, 40). However, Külz et al. (23) found that there were no significant differences between MBCT group (*n* = 61) and PE group (*n* = 64) after 8 weeks intervention in Y-BOCS. The participants in this study were OCD with residual symptoms after at least 20 sessions CBT, so it indicated that the importance of cognition aspect in the intervention. People who are not responded to CBT may also get little improvement by MBCT, which emphasized that the MBCT for OCD program cannot just adds mindfulness exercises into CBT, it should be an integration with its own conceptualization. Moreover, the same authors reported that the difference between groups became significant in the self-report instrument (OCI-R), and they thought it suggested that MBCT may help OCD to be more acceptable and validated which is more sensitive in OCI-R. This is consistent with our hypothesis that mindfulness training is able to develop and stabilize mental states that are incompatible with the mental states stimulated in OCD patients has been validated. We considered that the therapeutic efficacy of MBCT on OCD may result from neutralizing the cognitive biases and mechanisms of OCD, to shift from the affect regulation systems and dysfunctional responses to acceptance and non-response.

For the long-term efficiency, our results showed that the efficacy of MBCT intervention on OCD patients maintained until the 6-month follow-up, which was similar to that of SSRIs. However, the dropout due to loss of contact and the frequency and compliance of practicing mindfulness skills after MBCT, which may influence the analysis and cause bias in the long-term result were important factors. We did not detect significant differences in treatment responses at follow-up period between PE and the other two groups, which was consistent with previous research (23). It may be attributed to a placebo effect and group supportive influence, especially the setting of 10 weeks of PE group, aiming to be consistent with MBCT program. Secondly, although randomization was conducted, baseline anxiety and depression symptoms in PE group were milder than those in other groups, and the conclusion still exited after covariate analysis for balancing. Thirdly, dropout cases and two reporting AEs in PE group may influence the results. Furthermore, MBCT and PE were applied by the same treatment group, aiming to avoid therapist bias, but this may also influence the results in PE group regardless of the randomization and supervision.

Consistent with a previous study (24), MBCT interventions significantly improved depression, anxiety and quality of life, which are predisposing, activating and maintaining factors of OCD. There was a study compared MBCT with stress management training (SMT) in treating OCD, which also showed the advantages in relief to certain dysfunctional beliefs and stress (41). Interestingly, the mindfulness level of OCD patients in the three groups all increased at the same pace, although those in SSRI and PE groups never received mindfulness therapy. Further analysis indicated that the increased level of mindfulness was correlated to reduced Y-BOCS scores, indicating that a high level of mindfulness was beneficial to alleviate OCD symptoms, and in turn, remission of OCD symptoms increased mindfulness level.

Compared with the 20–25% dropout rate for CBT-ERP (5) and 26.83% of that for SSRIs, MBCT showed a good compliance, which supported the suggestion that mindfulness-based treatment for OCD might reduce dropout rate or attrition in psychological therapy (23, 42). It is considered that the high dropout rate for ERP is related to the exposure leading to a high level of anxiety, which cannot be well-tolerated by individuals suffering from OCD. On the other hand, mindfulness practice is an effective form of exposure because it helps individuals intentionally face their own thoughts, emotions and sensations as they arise rather than reacting to them (9). Instead, they learn to accept those thoughts, emotions, and sensations for the harmless and impermanent mental events they are handling with. Therefore, the combination of mindfulness-based treatment with traditional psychotherapy may be effective in changing the symptoms and enhancing treatment compliance. Furthermore, our results showed that MBCT treatment efficacy was not influenced by involved patients and therapists, and time points. Thus, we considered that the 10-week MBCT in the treatment of OCD is well acceptable and safe, which should be popularized in clinical application.

### Limitations

There were still some confounders in this study. The study sample was not good enough, which included more men than women overall and that baseline scores for anxiety and depression in the PE group were still lower than those in the other two groups even after the randomization. Participants in PE program also got 10 weeks intervention, which may result in more improvement than normal 8 weeks psycho-education intervention. These factors may contribute to the bias of the results, heightened the improvement of PE. But still, the outcome of PE was beyond our expectation because we considered that PE was only an active placebo generally presenting a supportive role. Also, the significant difference between intervention groups (MBCT and SSRIs) and placebo group (PE) was only showed in the comparison of clinical response, but not in the repeated measures ANOVA of scores. So that, the specific function of PE should be future investigated. Also, for the SSRIs group, this study limited the dose and range of medication administered, but did not record the process of medication adjustment for each participant in detail, which may be relevant to the efficacy of the medication. In future studies, medication administration in the SSRIs group needs to be included in a more detailed analysis.

Another limitation in the present study is that we used the active placebo as the comparison. In the future research, the therapeutic efficacy of MBCT on OCD should be compared with other first-line interventions like CBT. In addition, OCD patients with severe symptoms or other psychiatric conditions were excluded from this study, and these certain population needs to be concerned. We conjecture that medication may be more effective for more severe OCD and that the gap would be more significant in the PE group, but given the ethical issues, using CBT as a control group would be a more appropriate choice.

And the long-term effects of MBCT on OCD patients like the subsequent practice require more research. Further study should collect detail data about the frequency and duration of mindfulness practices beyond the sessions. Meanwhile, group support factors and practice conditions may also be benefit to the treatment of OCD, and in this study, the patients from SSRIs cannot get the group support compared with those from MBCT and PE, which may lead to a relatively less significant effect of the medicine, so in-depth exploratory researches for the specific effectiveness factors of MBCT for OCD should be conducted in the future.

Moreover, the DSM-5 removed obsessive-compulsive disorder from the category of anxiety disorders and defined it as a separate spectrum, and we also know that there are many different subtypes of OCD, but in this study we did not segment the different types of OCD for analysis. So whether the MBCT for OCD program, or a mindfulness-based intervention, would be effective for other disorders with similar symptom problems on the spectrum and whether it has different effects for different subtypes of OCD are worth further exploration in next studies and can help us to clarify more about the mechanisms by which the intervention works.

## Conclusions

In conclusion, MBCT could be considered as a novel intervention of unmedicated OCD patients with mild to moderate symptoms. In terms of the treatment response, the efficacy of MBCT on alleviating primary symptoms—obsession and compulsion, and secondary symptoms—anxiety and depression of these OCD patients is equal to SSRIs and better than PE. But the advantages are not significant in terms of the scale scores.

Also, the MBCT for OCD program can improve mindfulness level and it is suitable to be applied in clinical practice, which is acceptable and safe. In the future, we will continue to explore novel psychotherapy approaches to the treatment of OCD.

## Data Availability Statement

The raw data supporting the conclusions of this article will be made available by the authors, without undue reservation.

## Ethics Statement

The studies involving human participants were reviewed and approved by the Ethics Committee of the Shanghai Mental Health Center (SMHC). The patients/participants provided their written informed consent to participate in this study.

## Author Contributions

TZ was responsible for the conduct of research. LL was contributed to the adaption of MBCT and PE intervention program. FD helped with the MBCT for OCD program and supervision. ZW was the principal of quality control to medicine use. QF was in charge of quality control. HZ was responsible for supervision and review of the whole research. All authors contributed to the article and approved the submitted version.

## Conflict of Interest

The authors declare that the research was conducted in the absence of any commercial or financial relationships that could be construed as a potential conflict of interest.

## Publisher's Note

All claims expressed in this article are solely those of the authors and do not necessarily represent those of their affiliated organizations, or those of the publisher, the editors and the reviewers. Any product that may be evaluated in this article, or claim that may be made by its manufacturer, is not guaranteed or endorsed by the publisher.
